# A Case of Sciatica Terminating in Death

**Published:** 1891-07

**Authors:** T. M. Holmes

**Affiliations:** Rome, Ga.


### A CASE OF SCIATICA TERMINATING IN DEATH.

BY T. M. HOLMES, M. D., ROME, GA.

Read before the Medical Association of Georgia, Forty-Second Annual Session, Augusta, Georgia, April, 1891.

G. W. F. L., æt, fifty-five years, height six feet, complexion fair, weight about 160 pounds, married after thirty years of age, was the father of five children, had always been temperate, had for many years been a much respected citizen of Floyd county, and had always led an active public life ; and having for the last ten or fifteen years been a real estate agent, he did a great deal of walking. His residence was on a high hill, and he had to climb this hill three or four times a day. It was in the summer of 1889 that he consulted me first concerning a soft, round tumor, about the size of a partridge egg, on the anterior and inferior aspect of the right thigh, just over the lower corner of scarpa's triangle. The tumor had been growing for several months, was of a dark chocolate color, and was attached by a short, thick pedicle, and the edges of the tumor were overhanging and lay close to the surface of the thigh. In general outline it was probably more analagous to the large warts frequently seen on cattle, but in color it was more like a mole. I advised treating it once a day with strong nitric acid, which he did, and in about two weeks it dropped off, leaving a perfectly smooth, but black pigmented surface. It never returned, but in about six months after its removal the patient called at my office to show me an enlarged lymphatic gland, lying in the second interspace, about two inches from where the eight pictorails major attaches to the humerus. It was about a half an inch in length and a quarter of an inch in diameter, fusiform in shape, and was tender from constant rubbing of the clothing. For this I prescribed iodine externally and iodide of potash, bicloride of mercury, Fowler's solution of arsenic and compound tincture of gentian, as an alterative and tonic, but despite its constant use the tumor gradually enlarged, so that I advised its excision. To this the patient agreed, and on July 20, 1890, I injected a drachm of a four per cent. solution of cocaine, and after making a sufficient incision, I removed the tumor by tearing, thus making an almost bloodless operation. At the time of its removal, the tumor was fully an inch in diameter. The wound was thoroughly cleansed with a $\frac{1}{2000}$ bichloride solution, and the edges brought together with antiseptic silk sutures, and the whole dressed with antiseptic lint. The arm was worn in a sling, so that the parts were kept perfectly at rest, but despite these antiseptic and precautionary measures the wound did not heal entirely for quite a month, and principally by granulation. After this, things went well for several months, when other glands near the right axilla, around the posterior cervical region and in the lower intercostal spaces, began to hypertrophy, one or two of them becoming half as large as a guinea egg. Recourse was again had to the constitutional treatment and local applications, and in this way they were held in check and really decreased in size. When the treatment was left off at any time longer than a fortnight, they would begin to increase in size and number perceptibly. By the use of those remedies they were held in check for many months, and the outlook seemed to be that the old man could safely count on two or three years longer before the disease, which I knew to be lymphadenoma, would steal his strength and life away. He had had considerable mental anxiety about his condition, because he had seen two of his old friends die of the same disease. Their pictures were ever before him, with their hideous lymphatic tumors larger than a man's fist, in perfect pones around their necks, choking their lives away, and he thought himself in a similar condition. My purpose was to ward off this condition as long as possible, for neither he nor I ever suspected that the scythe of time was soon to mow him down with another blade than the one in view.

On the morning of October 5th, 1890, I was called to see him for a very excruciating neuralgia in his right leg, extending from the hip to the foot over the course of the sciatic nerve and down into the calf. He had been having cramping spells, or spasmodic pains, in the calves of his legs for several weeks, which were very intense, coming on without a moment,s warning while he was sitting in his office or walking the streets, and the only way in which relief could be had was by grasping the calf in his hands and squeezing tightly. This would overcome the spasm and the pains would subside. Their favorite hour for coming on was about 3 o'clock in the morning, when they would be so intense as to make him lie and cry aloud so agonizingly as to arouse his whole family and be heard several hundred yards away. This was the condition in which I found him the morning of October the 5th, at which time I gave him a quarter of a grain of sulphate of morphine, injected into the seat of pain, and wrapped the parts in flannel cloths, saturated in chloroform liniment. This relieved him until the same hour next morning, when the pains recurred as bad as before. The same treatment, too, was less successful, in that it did not control the pains but for a few hours. After this I found it necessary to give the hypodermics of morphine several times during the twenty-four hours, and I also discovered that pains of the same character, only in a milder degree, existed on the left side. He did not complain of this side, however, but very little. Owing to the periodicity that seemed to characterize the pains, I put him on five grain doses of quinine every three hours till twenty-five grains were taken each day, and pursuing this course of treatment for several days, without any good results, I abandoned it and substituted therefor iron, arsenic and strychnine. At the same time I tried time and again to get a blister over the whole length of the nerve, but owing to the anesthesia, or low vitality of the parts, no cantharidal ointment, however good, would make an impression. I then tried the constant faradic current which gave momentary relief; and I then tried injections of chloroform into the nerve sheath, the effect of which seemed to be beneficial as to the relief of pain. Feeling that it was necessary to cleanse the alimentary canal of all fecal lumps I gave him castor oil several times, which always made him deathly sick. His bowels were very much constipated, and his appitite was almost *nil.* The bowels were evidently in a very atonic condition, being probably at this time in a paretic state. Within about ten days from the time I was first called the pain began to leave his legs from below, and as it receded total paralysis of motion ensued to as high up as the umbilical region. For about two weeks after paralysis of motion, total paralysis of sensation also took place, but only from about the lower third of the thighs downwards, but after that it extended gradually over the entire area of motor paralysis. The patient was now perfectly helpless, so far as the lower half of his body was concerned, being unable to move the slightest or to avoid his urine or evacuate his bowels. In the upper half of his body he was as strong and active as when in perfect health. Pains of a greater or less severity would shoot through his abdominal region, and seemed to be both in his intestines and abdominal parietes. Having at this juncture called Dr. J. B. S Holmes in consultation, we agreed to give another dose of castor oil and still continue to remove the fecal lumps from his bowels. He was given a large dose, but we were never able to get a full and free evacuation, but the contents of the bowels were entirely softened and ran from him involuntarily up to his death, the 15th of November, or about six weeks after I was first called. He was perfectly conscious to the hour of his death, though he was for several days too weak to speak above a whisper.

At the time of his death he was a miserable skeleton, and a tremendous, nauseating bedsore extended across his posterior pelvic region. This was due to the fact that he could lie in no other position than on his back, because of excessive and persistent nausea when in any other position. The commingled odor from this sore and the ever-flowing alvine discharges made it very disagreeable to those around him, as well as to himself, and death, for which he constantly prayed, was a sweet relief. During the course of his illness several physicians saw h him, and all were impressed with the uniqeness of the case. That sciatica, pure and simple, should produce death was an unheard of thing, and yet it was difficult to say positively that the lymphatic trouble was a material factor in the production of the newmotic trouble. There were not a great many of the hyperteophied glands discernable, but that a greater number existed in the interior of the body was highly probable,and since the lymphatic system is one of the most important in the making of healthy blood, we can readily see how an anemic condition of the entire nervous system might naturally follow, but just why the lower half of the body alone should become neuralgic and paralyzed is difficult to explain.

